# Mesenchymal Stem-Cell-Derived Exosomes as Novel Drug Carriers in Anti-Cancer Treatment: A Myth or Reality?

**DOI:** 10.3390/cells14030202

**Published:** 2025-01-29

**Authors:** Carl Randall Harrell, Ana Volarevic, Valentin Djonov, Vladislav Volarevic

**Affiliations:** 1 Regenerative Processing Plant, LLC, 34176 US Highway 19 N, Palm Harbor, FL 34684, USA; dr.harrell@regenerativeplant.org; 2 Departments of Psychology, Center for Research on Harmful Effects of Biological and Chemical Hazards, Faculty of Medical Sciences, University of Kragujevac, 69 Svetozara Markovica Street, 34000 Kragujevac, Serbia; ana.volarevic@fmn.kg.ac.rs; 3 Institute of Anatomy, University of Bern, Baltzerstrasse 2, 3012 Bern, Switzerland; valentin.djonov@unibe.ch; 4 Departments of Genetics, Microbiology and Immunology, Center for Research on Harmful Effects of Biological and Chemical Hazards, Faculty of Medical Sciences, University of Kragujevac, 69 Svetozara Markovica Street, 34000 Kragujevac, Serbia; 5 Faculty of Pharmacy Novi Sad, Trg Mladenaca 5, 21000 Novi Sad, Serbia

**Keywords:** mesenchymal stem cells, exosomes, anti-cancer agents, micro RNAs, target therapy

## Abstract

Although cancer therapy has significantly advanced in recent decades, patients and healthcare professionals are still quite concerned about adverse effects due to the non-targeted nature of currently used chemotherapeutics. Results obtained in a large number of recently published experimental studies indicated that mesenchymal stem-cell-derived exosomes (MSC-Exos), due to their biocompatibility, ability to cross biological barriers, and inherent targeting capabilities, could be used as a promising drug-delivery system for anti-cancer therapies. Their lipid bilayer protects cargo of anti-cancer drugs, making them excellent candidates for the delivery of therapeutic agents. MSC-Exos could be engineered to express ligands specific for tumor cells and, therefore, could selectively deliver anti-cancer agents directly in malignant cells, minimizing side effects associated with chemotherapeutic-dependent injury of healthy cells. MSC-Exos can carry multiple therapeutic agents, including anti-cancer drugs, micro RNAs, and small bioactive molecules, which can concurrently target multiple signaling pathways, preventing tumor growth and progression and overcoming resistance of tumor cells to many standard chemotherapeutics. Accordingly, in this review article, we summarized current knowledge and future perspectives about the therapeutic potential of MSCs-Exos in anti-cancer treatment, opening new avenues for the targeted therapy of malignant diseases.

## 1. Introduction

Cancer treatment has made significant strides over the past few decades, yet the side effects associated with the non-targeted nature of current therapeutic approaches remain a considerable concern for patients and healthcare providers [1]. Chemotherapy, one of the most common anti-cancer therapies, employs drugs that are designed to eliminate tumor cells [2]. However, these drugs do not discriminate between rapidly dividing malignant and healthy cells [1,2]. For instance, cells in the bone marrow, gastrointestinal tract, and hair follicles are also affected. This indiscriminate action can cause severe side effects, such as myelosuppression, leading to anemia, increased susceptibility to infections, and bleeding problems due to low erythrocyte, leukocyte, and platelet counts [2]. Nausea and vomiting are the main consequences of the chemotherapy’s negative impact on the viability of epithelial cells within the gastrointestinal tract [1,2]. Radiation therapy, while effective in targeting tumor sites, can damage surrounding healthy tissues, resulting in acute effects, such as skin irritation or burns, as well as long-term complications like fibrosis or organ dysfunction [3]. Patients may also experience fatigue, which is often exacerbated by the cumulative effects of both radiation and chemotherapy [3].

Newer targeted therapies, including monoclonal antibodies and small molecule inhibitors, are used to disrupt specific molecular pathways involved in cancer growth [4,5]. While these therapies generally have a more favorable side effect profile compared to traditional methods, they are not entirely free from adverse effects [4,5]. Infusion-related reactions, including fever, chills, and nausea, skin reactions (rashes and itching) and immune-related adverse events, such as colitis or thyroid dysfunction, may be observed in some patients who received monoclonal antibodies and small molecule inhibitors as anti-cancer agents [4,5]. The side effects of these targeted treatments highlight the need for the design and clinical use of new anti-cancer agents.

Results obtained in a large number of recently published experimental studies indicated that mesenchymal stem cell (MSC)-derived exosomes (MSC-Exos) could be used as novel drug carriers in anti-cancer treatment, reducing side effects of chemotherapeutics, radioactive compounds, and small molecule inhibitors [6,7,8,9,10,11,12,13,14]. Accordingly, in this review article, we summarize current knowledge about the therapeutic potential of MSCs-Exos in anti-cancer treatment, opening new avenues for the targeted therapy of malignant diseases. An extensive literature review was carried out in October 2024 across several databases (MEDLINE, EMBASE, Google Scholar) from 1990 to present. Keywords used in the selection were as follows: “anti-cancer treatment”, “chemotherapeutics”, “radioactive compounds”, “small molecule inhibitors”, “mesenchymal stem cell-derived exosomes”, “targeted therapy”, “signaling pathways”, and “tissue repair and regeneration”. All journals were considered and an initial search retrieved 142 articles. The abstracts of all these articles were subsequently reviewed by two of the authors (CRH and VV) independently to check their relevance to the subject of this manuscript. Eligible studies had to delineate molecular and cellular mechanisms that are involved in MSC-Exo-dependent delivery of anti-cancer agents in malignant cells, attenuating their side effects and improving their efficacy in the elimination of malignant cells.

## 2. MSC-Exos: Small Extracellular Vesicles with Huge Therapeutic Potential

MSCs are adult stem cells, which have garnered significant attention for their therapeutic potential in treating inflammatory and degenerative diseases due to their unique capacity to modulate immune response and to enhance viability and proliferation of injured parenchymal cells, promoting tissue repair and regeneration [15]. MSCs spontaneously generate cells of mesodermal origin (chondrocytes, osteoblasts, adipocytes) and have the ability to differentiate into the cells of endodermal and ectodermal origin when they grow under specific culture conditions [16]. MSCs secrete a variety of bioactive factors that can modulate migration, survival, phenotype and function of immune cells [17]. MSC-derived interleukin 1 receptor antagonist (IL-1Ra) and soluble receptors of tumor necrosis factor alpha (sTNFRs) suppress interleukin 1 (IL-1) and tumor necrosis factor alpha (TNF-α)-dependent recruitment of circulating leukocytes in inflamed tissues. MSC-sourced prostaglandin E2 (PGE2) and interleukin 10 (IL-10) promote the generation of alternatively activated macrophages and induce tolerogenic phenotype in dendritic cells (DCs), alleviating their antigen-presenting properties. MSC-derived transforming growth factor beta (TGF-β) inhibits proliferation of inflammatory Th1 and Th17 lymphocytes, while MSC-sourced indoleamine 2,3-dioxygenase (IDO) promotes expansion of immunosuppressive T regulatory cells (Tregs), enabling alleviation of ongoing inflammatory response [17]. Similarly, MSC-derived IL-10, PGE2, and TGF-β promote expansion of myeloid-derived suppressor cells (MDSCs), further contributing to the generation of the immunosuppressive environment in inflamed and injured tissues. Although the majority of MSC-derived factors inhibit ongoing inflammation, MSCs are not constitutively immunosuppressive cells [17]. MSCs adopt their phenotype and function according to the tissue microenvironment in which they were transplanted [18]. If MSCs are exposed to the elevated concentrations of inflammatory cytokines (interferon gamma (IFN-γ) and TNF-α), MSCs obtain immunosuppressive phenotype and secrete large amounts of immunosuppressive cytokines. However, when MSCs are exposed to the low concentrations of IFN-γ and TNF-α, they act as inflammatory cells and produce pro-inflammatory cytokines (TNF-α, IL-1β, IL-6, IFN-γ), which enhance antigen-presenting properties of DCs and macrophages, enabling increased generation and proliferation of inflammatory Th1 and Th17 cells [18]. Accordingly, MSCs which were transplanted in experimental mice 24 h after injection of tumor cells, obtained pro-inflammatory phenotype and promoted expansion of cytotoxic T lymphocytes, which resulted in attenuated tumor growth and progression [19]. However, MSCs which were transplanted 14 days after tumor induction were exposed to a high concentration of inflammatory cytokines and therefore obtained an immunosuppressive phenotype. These MSCs inhibited anti-tumor immune response and promoted tumor growth and progression [19].

The majority of MSC-derived immunomodulatory factors are present within MSC-Exos, small, spherical extracellular vesicles (EVs) [20]. Due to their nano-size (30–150 nm in diameter) and lipid envelope, MSC-Exos could easily bypass all biological barriers in the body and are, therefore, used in intercellular communication between MSCs and target cells (immune cells, endothelial cells (ECs), parenchymal cells) [20]. MSC-Exos are enriched in MSC-derived microRNAs (miRNAs) and MSC-sourced proteins involved in cellular signaling (such as heat shock proteins (HSP70, HSP90), tetraspanins (CD9, CD63, CD81)), immunomodulation (IL-10, TGF-β, Kynurenine, IL-1Ra, sTNFRs), proliferation (hepatocyte growth factor (HGF), neural growth factor (NGF), platelet-derived growth factor (PDGF)), and neo-angiogenesis (vascular endothelial growth factor (VEGF), angiopoietin) [21,22]. Accordingly, MSC-dependent regeneration of injured organs mainly relied on the capacity of MSC-Exos to promote survival of injured parenchymal cells and to induce enhanced proliferation and differentiation of progenitor cells and tissue-specific stem cells in damaged tissues [21,22]. MSC-Exos-dependent suppression of immune cell-driven inflammation was mainly responsible for the beneficial effects of MSCs in target therapy of inflammatory and autoimmune diseases [20,21]. Furthermore, due to their ability to deliver MSC-sourced pro-angiogenic factors in ischemic areas, MSC-Exos were used for re-vascularization of ischemic tissues as potentially novel therapeutic agents for the treatment of cerebrovascular and cardiovascular diseases [23].

In similar manner as their parental MSCs, MSC-Exos may promote or suppress tumor growth, depending on their content [24,25,26]. If MSC-Exos are derived from pro-inflammatory MSCs, they are enriched with inflammatory cytokines and favor the generation and expansion of cytotoxic lymphocytes and monocytes, contributing to the attenuation of tumor progression [25,26]. However, MSC-Exos, which are produced by immunosuppressive MSCs, contain anti-inflammatory and immunosuppressive factors and inhibit anti-tumor immunity, enabling the rapid growth of existing tumors [26]. Similarly, MSC-Exos, which are enriched with pro-angiogenic factors, enhance generation of new blood vessels and, in this way, promote tumor growth and metastasis [25,26].

Although MSC-Exos provide notable benefits in the therapy of inflammatory and ischemic diseases, they also come with certain disadvantages compared to using their parental MSCs [18,21]. One major drawback is the limited lifespan and quantity of MSC-Exos that can be harvested from cultured MSCs, which may hinder their therapeutic efficacy and necessitate repeated dosing [21]. Unlike MSCs, which can proliferate and be expanded in culture, MSC-Exos production relies on the parent cells’ health and viability [22]. There is also the risk of variability in MSC-Exos composition based on the source of MSCs, culture conditions, and isolation methods, leading to inconsistencies in treatment efficacy [18]. Lastly, while MSC-Exos can facilitate cell signaling and tissue regeneration, they lack the direct differentiation potential and functional roles that whole MSCs can provide in certain therapeutic contexts, limiting their applicability for specific conditions that require cellular repair rather than just signaling [18,21].

However, it should be also emphasized that there are several advantages for using MSC-Exos instead of MSCs in regenerative medicine [20,21]. Firstly, MSC-Exos are significantly smaller and more stable, allowing their easier delivery through various routes, including intravenous administration, which enhances their therapeutic potential and efficacy [21]. Additionally, MSC-Exos do not require the same level of complex culture and maintenance conditions as MSCs, simplifying manufacturing processes and lowering costs [18,20]. Finally, their ability to cross biological barriers, including the blood–brain barrier, further expands their potential applications in treating neurological conditions [20,21].

## 3. MSC-Exos: Novel, Nano-Sized Carriers of Anti-Tumor Drugs

In order to reduce the risk of MSC-Exos-dependent promotion of tumor growth, many research groups decided to modify MSC-Exos and to use them as acellular drug-delivery systems [6,7,8,9,10,11,12,13,14]. This modification process includes incorporating anti-cancer drugs into the exosomes and adjusting their surface charge to enhance drug uptake [6,27]. A variety of loading techniques have been developed to improve the efficacy of exosomes in cancer treatment. These strategies are generally classified into two main categories: indirect methods involving endocellular loading and direct methods involving extracellular loading [6,27,28]. Endocellular loading typically employs methods like co-incubation or genetic modification to introduce genes or cargo into the exosomes. In this way, the therapeutic cargo is delivered into the parental MSCs. Once the cargo is encapsulated within the MSC-Exos, these exosomes are harvested for therapeutic use [6,27]. Conversely, extracellular loading methods involve directly adding cargo to exosomes that have been isolated from MSCs using active techniques such as electroporation, sonication, incubation, and freeze–thaw cycles, or through passive techniques like surface modification, hybridization, and biomimetic approaches [6,27,28].

Various loading methods were used for the loading of different anti-cancer agents (Table 1) [29,30]. Gomari and colleagues successfully implemented an electroporation technique to create temporary hydrophilic pores in the MSC-Exos’ membrane, enabling incorporation of doxorubicin into MSC-Exos [29]. In vitro obtained results showed that cytotoxicity of doxorubicin-loaded MSC-Exos against HER2/neu-overexpressing TUBO breast cancer cells was higher than the cytotoxicity of free doxorubicin. Similar findings were observed in a murine breast cancer model. Doxorubicin-loaded MSC-Exos accumulated in the target tumor tissue more efficiently than free doxorubicin reduced breast cancer growth [29]. Kamerkar and colleagues utilized electroporation to introduce short interfering RNAs (siRNAs) and short hairpin RNA (shRNA) in MSC-Exos [30]. These RNAs specifically targeted Kras^G12D^ mutation, which is responsible for the production of Kras protein that promotes uncontrolled proliferation of tumor cells, playing a crucially important role in the development of pancreatic ductal adenocarcinoma (PDAC) [31]. By targeting Kras^G12D^ mutation, siRNA and shRNA-loaded MSC-Exos suppressed the growth and progression of cancer cells in mouse models of PDAC and significantly increased overall survival of MSC-Exos-treated PDAC-bearing experimental animals [30].

Hosseini and colleagues recently demonstrated that Wharton’s Jelly mesenchymal stem-cell-derived exosomes (WJ-MSC-Exos) could be used as nano-sized carriers of STAT-3 inhibitor (S3I-201) in the therapy of triple-negative breast cancer (TNBC), a rare form of invasive breast cancer [32]. By delivering S2I-201 in TNBC cells, WJ-MSC-Exos down-regulated expression of anti-apoptotic proteins (Bcl-2 and p-STAT3), increased activity of pro-apoptotic proteins (Bax and caspase-3), inducing apoptosis of malignant cells. Growth of TNBC was significantly more reduced in tumor-bearing animals that received S3I-201-loaded WJ-MSC-Exos compared to S3I-201-treated TNBC-carrying animals, suggesting that WJ-MSC-Exos significantly enhanced the therapeutic efficacy of S3I-201 in the treatment of TNBC [32].

Similar results were obtained by Abas and colleagues who demonstrated that WJ-MSC-Exos could serve as a drug-delivery system for paclitaxel-based therapy of cervical cancer [33]. It is well known that, after a certain period of treatment, cervical cancer cells developed resistance to paclitaxel and underwent epithelial–mesenchymal transition (EMT), obtaining more potent migratory and invasive properties [34]. To avoid resistance of cancer cells, paclitaxel should be given at a lower dose and transported into cancer cells with a more specific delivery system [34]. Using the method of electroporation, Abas and colleagues successfully loaded paclitaxel in WJ-MSC-Exos and demonstrated efficacy of these Exos against Hela cervical cancer cells [33]. When paclitaxel was delivered by WJ-MSC-Exos, it induced Bax and caspase-3-driven apoptosis of Hela cells at lower concentrations than free paclitaxel and more efficiently inhibited Notch and TGF-β-dependent EMT, attenuating invasiveness of cervical cancer cells [33].

Clinical application of vincristine sulfate (VCR sulfate) is restricted due to its severe side effects, which are consequences of VCR sulfate’s non-specific targeting and toxic damage of healthy, non-malignant cells [35]. Farouk and colleagues used MSC-Exos to deliver VCR sulfate in breast cancer cells [36]. Despite the fact that the cytotoxicity of VCR sulfate-loaded MSC-Exos against tumor cells was nearly the same as that of free VCR sulfate, MSC-Exos significantly improved targeted delivery of VCT sulfate and lessened its side effects [36].

Valepour and colleagues used human endometrial MSC-Exos (hEnd-MSC-Exos) as carriers for atorvastatin and investigated their pro-apoptotic effects against glioblastoma cells [37]. Atorvastatin-loaded hEnd-MSC-Exos enhanced expression of pro-apoptotic Bax and suppressed synthesis of anti-apoptotic Bcl-2 protein in tumor cells more efficiently than atorvastatin alone [37]. Additionally, a significant decrease in VEGF secretion was observed when glioblastoma cells were co-cultured with human-umbilical-vein endothelial cells (HUVECs) in the presence of Atorvastatin-loaded hEnd-MSC-Exos, suggesting their capacity for angiomodulation in the tumor microenvironment [37].

Norcantharidin (NCTD) demonstrates significant therapeutic potential in the treatment of hepatocellular carcinoma (HCC) [38]. NCTD induces apoptosis and inhibits cell proliferation through the modulation of PI3K/Akt and MAPK signaling [38]. However, its clinical application is limited by severe side effects, such as hepatotoxicity and gastrointestinal disturbances, which necessitate careful monitoring and optimization of dosing protocols [39]. Liang and colleagues loaded NCTD into BM-MSC-Exos via electroporation and evaluated their effects against HCC cells [40]. BM-MSC-Exos provided targeted, continuous, and slow delivery of the NCTD. Compared with the NCTD treatment alone, BM-MSC-Exos-NCTD-based therapy more efficiently induced cell-cycle arrest and apoptosis of HCC cells and more effectively attenuated growth and progression of liver cancer in experimental animals, suggesting that MSC-Exos could be considered for a drug-delivery system in anti-cancer therapy [40].

**Table 1:** MSC-Exos-mediated delivery of anti-cancer drugs and bioactive molecules.

Tissue Source of MSC-Exos	Incorporated Drug or Bioactive Molecule	Loading Technique, Delivery Method or Genetic Modification of MSC-Exos	Target Tumor Cells	Biological Effects	Ref. No.
MSC-exos-mediated delivery of chemotherapeutics					
Bone marrow	Doxorubicin	Electroporation	HER2/neu-overexpressing TUBO breast cancer cells	Increased toxicity of doxorubicin against cancer cells; Enhanced accumulation in the tumor tissue	[29]
Skin	Kras^G12D^—specific siRNAs ad shRNAs	Electroporation	Pancreatic ductal adenocarcinoma cells	Suppressed growth and progression of cancer cells; increased survival of MSC-Exos-treated tumor-bearing animals	[30,31]
Wharton’s Jelly	STAT-3 inhibitor (S3I-201)	Electroporation	Triple-negative breast cancer cells	Increased apoptosis of tumor cells; Reduced tumor growth and progression	[32]
Wharton’s Jelly	Paclitaxel	Electroporation	Hela cervical cancer cells	Increased apoptosis, attenuated EMT and invasiveness of cancer cells	[33]
Bone marrow	Vincristine sulfate	Sonicaton	Breast cancer cells	MSC-Exos improved targeted intra-tumor delivery of vincristine sulfate and lessen its side effects	[36]
Endometrium	Atorvastatin	Electroporation	Glioblastoma cells	Increased apoptosis of tumor cells	[37]
Bone marrow	Norcantharidin	Electroporation	Hepatocellular carcinoma cells	Increased cell-cycle arrest and apoptosis of cancer cells; Attenuated growth and progression of liver cancer in experimental animals	[40]
MSC-Exos-based delivery of oncolytic viruses					
Umbilical cord	Reovirus	Clathrin-mediated endocytosis and macropinocytosis	Acute myeloid leukemia cells	Direct oncolytic effect against tumor cells; enhanced anti-tumor immune response	[41,42]
MSC-Exos-dependent delivery of miRNAs					
Bone marrow	MiR-29b	MiR-29b-integrating recombinant lentiviral vector	Gastric cancer cells	Reduced number of peritoneal metastasis in experimental animals	[43]
Bone marrow	Antisense microRNA oligonucleotide	Electroporation	Glioblastoma cells	Attenuated tumor growth and progression in experimental animals	[44]
Adipose tissue	MiR-138-5p	MiR-138-5p-integrating recombinant lentiviral vector	Bladder cancer cells	Impaired proliferation, migration, and invasiveness of tumor cells; Reduced tumor growth in experimental animals	[45]
Umbilical cord blood	MiR-26a	Overexpression of anti-Glypican 3 single-stranded variable fragment and electroporation of miR-26a	Hepatocellular carcinoma cells	Attenuated proliferation and invasiveness of tumor cells; reduced growth and progression of liver cancer in experimental animals	[46]
Umbilical cord	Anti-Mir221	Overexpression of iRGD-Lamp2b	Colon cancer cells	Suppressed growth and progression of colon cancer in experimental animals	[47]

## 4. MSC-Exos-Mediated Delivery of Oncolytic Viruses

In addition to chemotherapy, oncolytic viruses, like reovirus, could serve as an effective treatment option for acute myeloid leukemia (AML). Oncolytic viruses are live viruses capable of replicating, specifically within cancer cells. When these viruses infect cancer cells, it leads to cell lysis, releasing additional viral particles into the nearby tissue. The newly produced viral particles can then infect adjacent cancer cells. With each cycle of infection, replication, lysis, and release, the virus can proliferate and disseminate throughout the tumor, potentially eliminating the entire tumor mass [48]. However, effectiveness of intravenously delivered reovirus is reduced because of the activity of neutralizing antibodies in the bloodstream [41]. Reovirus-specific antibodies bind to the epitopes on the membrane of viral antigens, preventing their entrance in the target tumor cells [41]. Additionally, the effectiveness of oncolytic viruses stems not only from their direct ability to destroy cancer cells but also from their capacity to trigger an anti-tumor cytotoxic adaptive immune response [48]. By using retrovirus loaded umbilical-cord-derived MSC-Exos, Yang and colleagues avoided antibody-dependent anti-viral immune response and successfully delivered retrovirus in tumor cells through clathrin-mediated endocytosis and macropinocytosis [42]. UC-MSC-Exo-delivered reovirus had a direct oncolytic effect on AML cells. Additionally, reovirus-infected AML cells were recognized by DCs, which presented viral antigens of AML cells to CD8+ cytotoxic T cells (CTL), inducing the activation of CTL-driven immune response against AML cells [42].

These results suggest that the combination of oncolytic viruses and MSC-Exos presents a promising avenue for cancer therapy, leveraging the unique properties of both to enhance treatment efficacy [41]. It should be emphasized that the packaging process of oncolytic-virus-loaded MSC-Exos is critical for their therapeutic application [49]. MSC-Exos can effectively encapsulate viral particles, aiding in targeted delivery to tumor cells [42,49]. This encapsulation not only enhances the stability of the viruses but also improves their accumulation in tumor sites due to the natural tumor-homing abilities of MSC-Exos [49,50]. The encapsulation of viral elements within exosomes facilitates prolonged circulation time and protects the virus from neutralizing antibodies, enhancing therapeutic outcomes [41]. However, it should be noted that tumor cells produce immunosuppressive cytokines (IL-10, IL-35 and TGF-β) [51]. An increased concentration of these immunosuppressive cytokines within the tumor microenvironment may inhibit the activation of the immune responses that oncolytic viruses are designed to stimulate [52]. Additionally, there are several challenges in manufacturing virus-associated MSC-Exos for anti-tumor therapy [49,50]. The tumor microenvironment often includes dense extracellular matrices that hinder the effective MSC-Exos-dependent delivery of oncolytic viruses [52]. Varying cellular characteristics of different tumors may affect the uptake and efficacy of MSC-Exos-delivered viruses [50,51]. Accordingly, different cancer cells may respond differently to MSC-Exos:oncolytic-virus-based treatment [49,50]. Finally, manufacturing oncolytic-virus-associated MSC-Exos at a large scale while maintaining consistent quality and functionality is a significant challenge [50]. This includes ensuring that the MSC-Exos are uniformly loaded with the virus and that they retain their biologically active forms [50]. Therefore, addressing the challenges of packaging efficiency, tumor microenvironment interactions, and immune evasion is critical for the successful application of MSC-Exos-dependent delivery of oncolytic viruses in anti-cancer treatment [42,50]. Ongoing research and development are essential to overcome these barriers and fully realize the potential of oncolytic-virus-associated MSC-Exos in clinical settings [51].

## 5. MSC-Exo-Dependent Delivery of Anti-Cancer miRNAs in Malignant Cells

MSC-derived miRNAs represent a group of small non-coding RNAs that regulate gene expression by targeting mRNA [53]. Several research groups used genetic engineering to obtain miRNA-overexpressing MSCs and to use their Exos for targeted delivery of miRNA in tumor tissues [53].

Kimura and colleagues transfected human BM-MSCs with miR-29b-integrating recombinant lentiviral vector, significantly increasing production of miR-29b [43]. MiR-29b down-regulates various genes associated with tumor progression and metastasis, such as those involved in EMT. By inhibiting these genes, MSC-derived miR-29b can help maintain the epithelial characteristics of cancer cells, reducing their invasiveness [54]. MSC-Exos, which were, by ultracentrifugation, isolated from culture supernatants of miR-29b-overexpressing BM-MSCs, contained markedly increased amounts of miR-29b compared with negative controls [43]. Results obtained in a murine model of gastric cancer showed that intraperitoneal transfer of miR-29b-carrying BM-MSC-Exos significantly reduced the number of peritoneal metastases in the mesentery and the momentum of experimental animals, suggesting that BM-MSC-Exos could be considered a useful carrier of anti-cancer miR-29b in the therapy of gastric cancer [43].

MiR-21 is highly expressed in glioblastoma, facilitating tumor growth by blocking the expression of apoptosis-related genes [55]. Therefore, an antisense microRNA oligonucleotide (AMO) against miR-21 was suggested as a therapeutic nucleic acid for glioblastoma [44]. Since MSC-Exos are able to easily bypass the blood–brain barrier (BBB), Kim and co-workers used them to deliver AMO directly into glioblastoma cells, efficiently attenuating glioblastoma growth and progression in experimental animals [44].

Liu and colleagues used adipose-tissue-derived MSC-Exos (AT-MSC-Exos) to deliver miR-138-5p in bladder cancer cells [45]. MiR-138-5p plays a critical role in the suppression of bladder cancer by inhibiting the expression of oncogenic YY1 factor, thereby reducing YY1-driven proliferation of cancer cells [56]. Additionally, miR-138-5p diminishes the migratory and invasive capabilities of bladder cancer cells by down-regulating vimentin and other EMT-related proteins. Accordingly, by delivering miR-138-5p directly in bladder cancer cells, AT-MSC-Exos impaired proliferation, migration, and invasiveness of these tumor cells and significantly reduced bladder cancer growth in experimental animals [45].

Mahati and colleagues recently demonstrated that anti-Glypican 3 (GPC3) single-stranded variable fragment (scFv)-modified MSC-Exos could be used for the delivery of anti-cancer agents in GPC3-expressing tumors, like GPC3-expressing HCC [46]. With the anti-cancer properties of miR-26a, which inhibits CDK6 and E2F3 oncogenes in HCC, in mind, Mahati et al. loaded miR-26a in anti-GPC3 scFv-expressing MSC-Exos and evaluated their therapeutic potential against HCC cells [46]. Anti-GPC3-scFv genetically modified MSC-Exos effectively delivered miR-26a into the GPC3-positive HCC cells and suppressed CDK6 and E2F3-dependent proliferation and invasiveness of tumor cells, attenuating growth and progression of HCC in experimental animals without causing any obvious side effects [46].

It is well known that anti-miR221 plays a pivotal role in combating cancer progression by antagonizing the effects of miR-221, which is overexpressed in many solid tumors [57]. By inhibiting miR-221, anti-miR221 restores the expression of its target tumor-suppressor genes, Phosphatase and Tensin Homolog (PTEN) and p57KIP2, both of which are critical for regulating cell growth and apoptosis. In this way, anti-miR221 inhibits cell proliferation and promotes programmed cell death of malignant cells, effectively slowing tumor progression [57]. In line with these findings, Han and colleagues loaded anti-miR221 into the human UC-MSC-Exos, which were genetically engineered to overexpress iRGD-Lamp2b [47]. The iRGD peptide is known for its tumor-targeting capabilities due to its ability to bind to integrins present on the surface of tumor cells and facilitate penetration into the tumor microenvironment. When conjugated with Lamp2b, a protein that aids in lysosomal targeting, iRGD enhances the delivery of therapeutic agents specifically to cancer cells [47]. In contrast to non-modified MSC-Exos which mainly accumulate in the lungs or liver of experimental animals after systemic infusion, intravenously injected iRGD-Lamp2b-overexpressing MSC-Exos mainly accumulated at tumor sites and successfully delivered anti-miR221 directly in colon cancer cells, efficiently suppressing tumor growth and progression [47]. These findings strongly indicate that iRGD-Lamp2b-overexpressing MSC-Exos should be further explored in clinical studies as potentially new delivery agents for intravenous infusions of anti-cancer drugs.

## 6. Current Challenges and Future Perspectives in MSC-Exo-Dependent Delivery of Anti-Cancer Agents

Despite the fact that a large number of experimental findings demonstrated that MSC-Exos could be used as vehicles for targeted delivery of anti-cancer agents, several challenges need to be addressed before these EVs could be widely used in clinical settings [6,21]. MSC-derived Exos can vary in heterogeneity and their capacity in loading therapeutic agents could be influenced by the tissue source of MSCs from which Exos were obtained [21,26].

One of the primary challenges in manufacturing MSC-Exos-based therapeutics is their isolation and purification of exosomes from biological fluids [58]. Traditional methods, such as ultracentrifugation, can be time-consuming and may lead to the co-isolation of contaminants like proteins, lipids, and other vesicles [58,59]. Techniques such as size-exclusion chromatography and precipitation have shown promise; however, they often lack the efficiency and scalability needed for clinical applications [58]. Developing robust and reproducible isolation protocols that yield high-purity MSC-Exos remains a critical hurdle for the field [21,26]. Another significant challenge is the characterization of MSC-Exos’ content, including profiling of exosomal proteins, lipids, and RNAs to ensure consistency and efficacy [58]. Advanced techniques like nanopore sequencing and mass spectrometry can provide insights into the molecular content of MSC-Exos, but they also require substantial resources and expertise, which can limit accessibility for smaller research facilities and companies [58]. As the demand for MSC-Exos-based therapies grows, the methods used to produce these vesicles must be scalable from laboratory to industrial levels without compromising quality [58,59]. Current cell culture systems, often used to generate MSC-Exos, may not produce sufficient quantities for therapeutic use [58,59]. Bioreactor systems that can support larger volumes while maintaining the requisite MSC-Exos’ quality are still in development. Furthermore, the variability in MSC-Exos yield and characteristics across different cell types and culture conditions complicates the establishment of a reliable manufacturing process [58]. Finally, regulatory considerations cannot be overlooked [26,58]. The Food and Drug Administration (FDA) and other regulatory agencies require stringent evaluation of therapeutics, including MSC-based products, which demands well-defined manufacturing processes and comprehensive safety assessments [26,58]. Developing guidelines specific to MSC-based therapeutics is necessary to facilitate their translation from bench to bedside [58]. This includes addressing concerns regarding immunogenicity, stability, and the long-term effects of exosome administration in patients [26,58].

In order to address these limitations, exosome mimetrics (EMs), artificially engineered nanoparticles, were designed [60]. Similar to natural MSC-Exos, EMs are composed of a phospholipid bilayer, which is crucial for encapsulating therapeutic agents and facilitating fusion with target cell membranes [61]. Inside the lipid bilayer, EMs can contain a core that may encapsulate various therapeutic agents, including proteins, RNAs, or small molecules. This core mimics the cargo of natural exosomes, allowing for targeted delivery [61]. EMs typically range from 30 to 150 nanometers in diameter, similar to the size of natural exosomes. Their spherical shape aids in stability and facilitates movement through biological fluids. Additionally, EMs can be engineered with various surface modifications, to enhance targeting specificity towards particular tumor cells, improving their therapeutic efficacy. Importantly, EMs offer several benefits as drug-delivery carriers compared to other artificial systems [61]. Firstly, phospholipid bilayers of EMs can merge with cell membranes, facilitating the uptake of the drugs they carry [62]. Additionally, the small size of these mimetics enhances their ability to pass through tumor blood vessels and infiltrate tumor tissue [61]. In line with these findings, by using mice model of osteosarcoma, Wang and colleagues recently demonstrated capacity of EMs to deliver anti-cancer agents in tumor cells without affecting neighboring healthy cells [63]. BM-MSCs were sequentially extruded to generate EMs. Afterwards, EMs were loaded with doxorubicin (BM-MSC-EM-Dox). Seven days after tumor induction, BM-MSC-EM-Dox (3mg/kg) were four times intravenously infused in experimental animals (every three days). Compared to free doxorubicin, an engineered BM-MSC-EM-Dox more efficiently inhibited tumor growth and induced fewer side effects in experimental animals [63].

## 7. Conclusions

**Figure 1:**
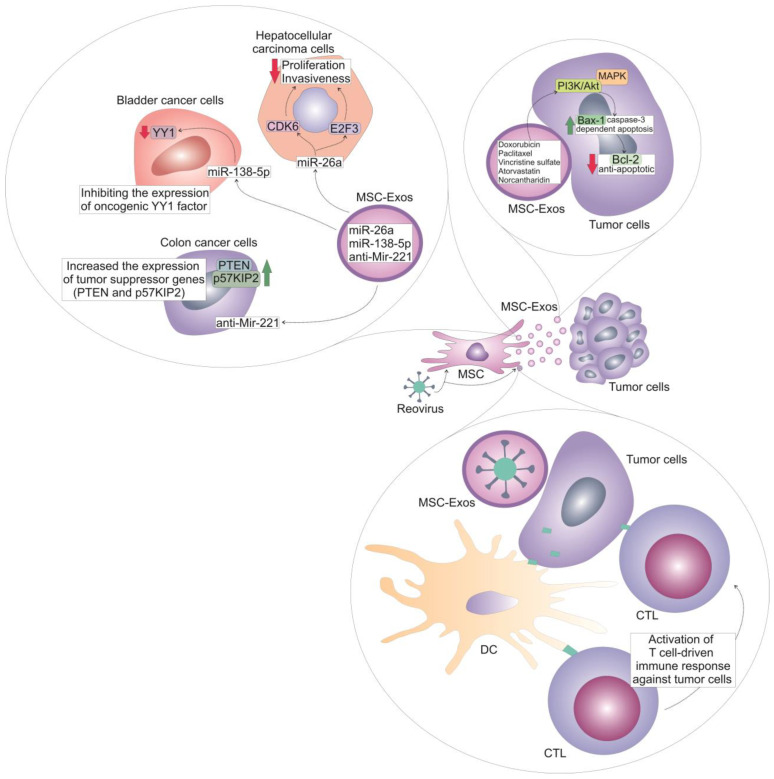
Molecular mechanisms responsible for the beneficial effects of MSC-Exos-mediated delivery of anti-cancer agents. Due to their nano-size dimension, biocompatibility, ability to cross biological barriers, and capacity to deliver their cargo directly into target cells, MSC-Exos have been used as carriers of many anti-cancer drugs and bioactive molecules. MSC-Exos were able to selectively deliver several chemotherapeutics (doxorubicin, paclitaxel, vincristine sulfate, atorvastatin, norcantharidin) in tumor cells, minimizing side effects associated with chemotherapeutic-driven injury of tumor-surrounding healthy cells. Additionally, MSC-Exos managed to increase accumulation of these anti-cancer drugs in tumor tissues and to enhance their toxicity against malignant cells. In tumor cells, MSC-Exos modulated PI3K/Akt and MAPK signaling pathways and induced programmed cell death by increasing synthesis of pro-apoptotic proteins (Bax and caspase-3) and by suppressing synthesis of anti-apoptotic Bcl-2 protein. By delivering reovirus directly in acute myeloid cells, MSC-Exos enhanced viral-induced oncolytic effects against these malignant cells. Additionally, reovirus-infected tumor cells were easily recognized by dendritic cells which presented viral antigens to cytotoxic T cells, inducing activation of T-cell-driven immune response against tumor cells. Additionally, MSC-Exos were used for targeted delivery of anti-cancer micro RNAs (miRNAs) in tumor tissues. By delivering miR-26a into hepatocellular carcinoma cells, MSC-Exos suppressed CDK6 and E2F3-dependent proliferation and invasiveness of tumor cells and inhibited rapid growth and progression of liver cancer in experimental animals. MSC-Exos were used to transfer miR-138-5p in bladder cancer cells, inhibiting the expression of oncogenic YY1 factor. MiR-29b-carrying MSC-Exos managed to significantly reduce total number of peritoneal metastases in gastric-cancer-bearing animals. By delivering anti-Mir221 in colon cancer cells, MSC-Exos increased the expression of tumor-suppressor genes (PTEN and p57KIP2), induced programmed cell death of malignant cells and attenuated growth and progression of colon cancer in experimental animals.

MSC-Exos should be considered as a promising drug-delivery system for anti-cancer therapies due to their biocompatibility, ability to cross biological barriers, and inherent targeting capabilities [10,11]. Their lipid bilayer protects the cargo, which can include anti-cancer drugs, nucleic acids, and small bioactive molecules, making them excellent candidates for delivering therapeutic agents [21,26]. MSC-Exos express majority of MSCs’ chemokine receptors and, therefore, retain the ability of their parental cells to home to tumor sites [7,8]. Within the tumor microenvironment, MSC-Exos may selectively deliver chemotherapeutics directly in target tumor cells, minimizing the side effects associated with chemotherapeutic-driven injury of tumor-surrounding healthy tissues [7,8] (Figure 1).

Importantly, MSC-Exos can be engineered to carry multiple therapeutic agents, including anti-cancer drugs, miRNAs, and small bioactive molecules [21,26]. In this way, MSC-Exos can concurrently target multiple signaling pathways responsible for cancer progression and may be used to overcome resistance of tumor cells to many standard chemotherapeutics [7,8].

The economic benefits of using MSC-Exos-based anti-tumor therapeutics compared to traditional anti-tumor strategies are becoming increasingly evident as research progresses [59]. MSC-Exos offer a more targeted delivery system that can enhance drug efficacy while minimizing systemic toxicity, potentially leading to reduced healthcare costs associated with managing side effects of conventional therapies like chemotherapy and radiation [26,59]. Additionally, the ability of MSC-Exos to carry a diverse range of therapeutic payloads, ranging from small molecules to RNAs, allows for more efficient treatment regimens, potentially shortening treatment duration and decreasing the need for multiple drugs [21]. Furthermore, advancements in manufacturing processes could lead to cost-effective production methodologies, making MSC-Exos-based therapies more accessible [58]. As clinical evidence mounts demonstrating improved patient outcomes and fewer complications, the long-term economic advantages of adopting MSC-Exos-based therapies over traditional methods could substantially outweigh initial investment costs, ultimately benefiting both healthcare systems and patients [59].

Therefore, it can be concluded that MSC-Exos represent a versatile and innovative strategy for drug delivery in cancer therapy, combining natural targeting capabilities with the potential for enhanced efficacy and reduced toxicity [26]. However, it should be noted that MSC-Exos can be sensitive to storage and handling conditions, which can lead to loss of their therapeutic potential [59]. Furthermore, determining optimal dosing regimens and effective delivery methods still remains a challenge, as it can vary widely depending on the target tumor tissue and the patient’s health conditions [21,26]. Challenges in manufacturing MSC-Exos-based therapeutics, such as characterization and profiling of their content, scalability, and regulatory considerations still need to be addressed before MSC-Exos-based drug delivery could become a mainstream therapeutic approach in oncology [58]. Accordingly, future experimental and clinical studies should address these challenges, paving the way for more effective MSC-Exos-based treatment of cancer patients.

In summation, therapeutic use of MSC-Exos for anti-tumor drug delivery is more a reality than a myth at this moment, backed by substantial scientific advancements and emerging clinical evidence. Promising results, obtained in various preclinical models, have demonstrated that MSC-Exos, with their natural ability to transport biologically active molecules and evade the immune system, could be engineered to efficiently deliver chemotherapeutic agents and anti-oncogenic miRNAs directly to tumor sites without causing severe side effects. Companies are now entering clinical trials, exploring exosome formulations for various cancers, signaling a shift from theoretical applications to tangible outcomes. The ongoing research and positive outcomes illustrate that the potential of MSC-Exos in anti-tumor drug delivery is not just a myth, but a burgeoning reality in the therapeutic landscape.

## Data Availability

The data that are discussed in this article are presented in cited studies.
